# Supplementary material for: Sexual Difference in the Optimum Environmental Conditions for Growth and Maturation of the Brown Alga Undaria pinnatifida in the Gametophyte Stage
Source: Genes (Basel). 2020 Aug 16;11(8):944. doi: 10.3390/genes11080944 (PMC7463851; doi:10.3390/genes11080944)
Supplement: Supplementary file 1 [file genes-11-00944-s001.zip › Supplemental Table2_REV.pptx]

## Slide 1
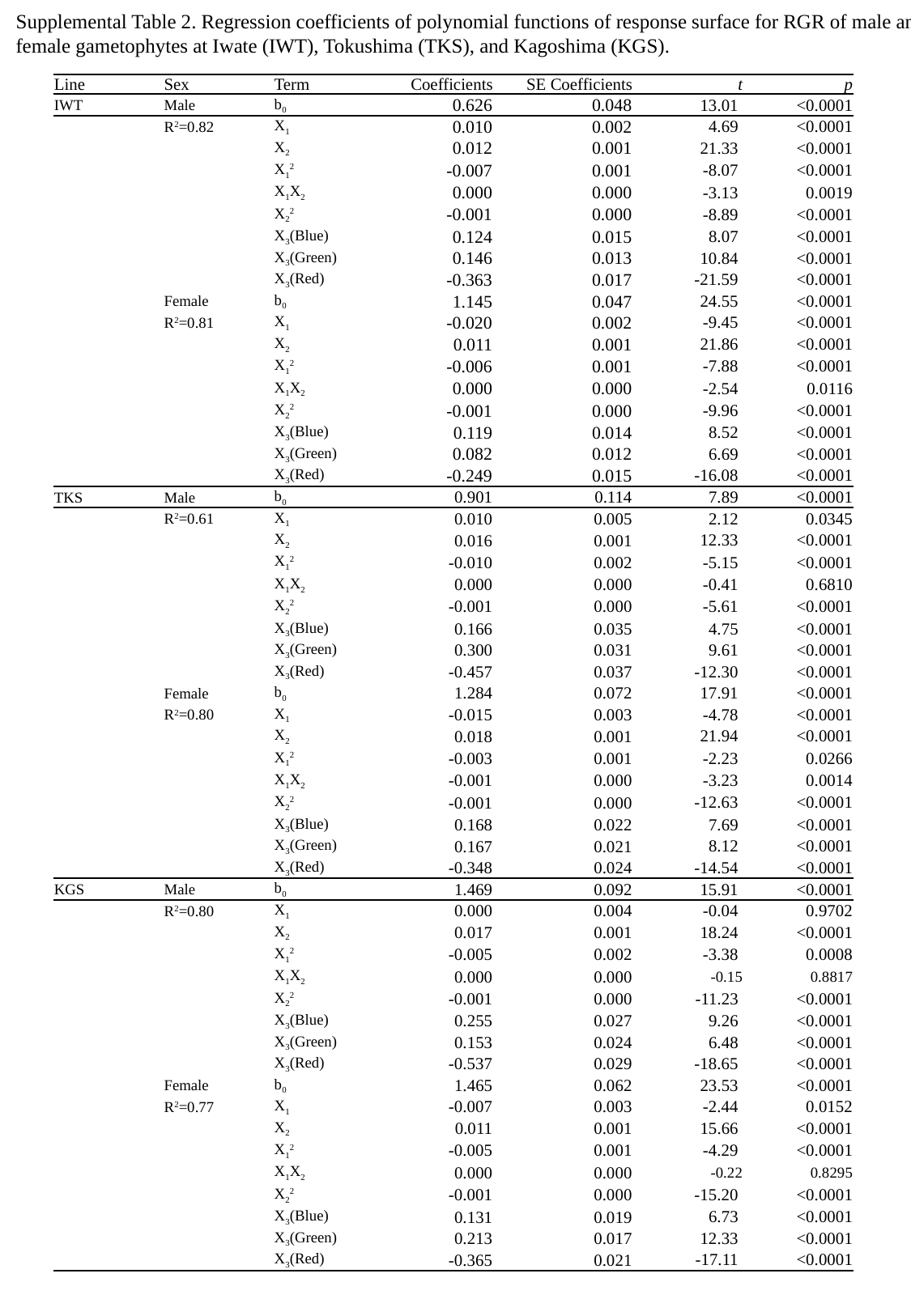

Supplemental Table 2. Regression coefficients of polynomial functions of response surface for RGR of male and female gametophytes at Iwate (IWT), Tokushima (TKS), and Kagoshima (KGS).
| Line | Sex | Term | Coefficients | SE Coefficients | t | p |
| --- | --- | --- | --- | --- | --- | --- |
| IWT | Male | b0 | 0.626 | 0.048 | 13.01 | <0.0001 |
| | R2=0.82 | X1 | 0.010 | 0.002 | 4.69 | <0.0001 |
| | | X2 | 0.012 | 0.001 | 21.33 | <0.0001 |
| | | X12 | -0.007 | 0.001 | -8.07 | <0.0001 |
| | | X1X2 | 0.000 | 0.000 | -3.13 | 0.0019 |
| | | X22 | -0.001 | 0.000 | -8.89 | <0.0001 |
| | | X3(Blue) | 0.124 | 0.015 | 8.07 | <0.0001 |
| | | X3(Green) | 0.146 | 0.013 | 10.84 | <0.0001 |
| | | X3(Red) | -0.363 | 0.017 | -21.59 | <0.0001 |
| | Female | b0 | 1.145 | 0.047 | 24.55 | <0.0001 |
| | R2=0.81 | X1 | -0.020 | 0.002 | -9.45 | <0.0001 |
| | | X2 | 0.011 | 0.001 | 21.86 | <0.0001 |
| | | X12 | -0.006 | 0.001 | -7.88 | <0.0001 |
| | | X1X2 | 0.000 | 0.000 | -2.54 | 0.0116 |
| | | X22 | -0.001 | 0.000 | -9.96 | <0.0001 |
| | | X3(Blue) | 0.119 | 0.014 | 8.52 | <0.0001 |
| | | X3(Green) | 0.082 | 0.012 | 6.69 | <0.0001 |
| | | X3(Red) | -0.249 | 0.015 | -16.08 | <0.0001 |
| TKS | Male | b0 | 0.901 | 0.114 | 7.89 | <0.0001 |
| | R2=0.61 | X1 | 0.010 | 0.005 | 2.12 | 0.0345 |
| | | X2 | 0.016 | 0.001 | 12.33 | <0.0001 |
| | | X12 | -0.010 | 0.002 | -5.15 | <0.0001 |
| | | X1X2 | 0.000 | 0.000 | -0.41 | 0.6810 |
| | | X22 | -0.001 | 0.000 | -5.61 | <0.0001 |
| | | X3(Blue) | 0.166 | 0.035 | 4.75 | <0.0001 |
| | | X3(Green) | 0.300 | 0.031 | 9.61 | <0.0001 |
| | | X3(Red) | -0.457 | 0.037 | -12.30 | <0.0001 |
| | Female | b0 | 1.284 | 0.072 | 17.91 | <0.0001 |
| | R2=0.80 | X1 | -0.015 | 0.003 | -4.78 | <0.0001 |
| | | X2 | 0.018 | 0.001 | 21.94 | <0.0001 |
| | | X12 | -0.003 | 0.001 | -2.23 | 0.0266 |
| | | X1X2 | -0.001 | 0.000 | -3.23 | 0.0014 |
| | | X22 | -0.001 | 0.000 | -12.63 | <0.0001 |
| | | X3(Blue) | 0.168 | 0.022 | 7.69 | <0.0001 |
| | | X3(Green) | 0.167 | 0.021 | 8.12 | <0.0001 |
| | | X3(Red) | -0.348 | 0.024 | -14.54 | <0.0001 |
| KGS | Male | b0 | 1.469 | 0.092 | 15.91 | <0.0001 |
| | R2=0.80 | X1 | 0.000 | 0.004 | -0.04 | 0.9702 |
| | | X2 | 0.017 | 0.001 | 18.24 | <0.0001 |
| | | X12 | -0.005 | 0.002 | -3.38 | 0.0008 |
| | | X1X2 | 0.000 | 0.000 | -0.15 | 0.8817 |
| | | X22 | -0.001 | 0.000 | -11.23 | <0.0001 |
| | | X3(Blue) | 0.255 | 0.027 | 9.26 | <0.0001 |
| | | X3(Green) | 0.153 | 0.024 | 6.48 | <0.0001 |
| | | X3(Red) | -0.537 | 0.029 | -18.65 | <0.0001 |
| | Female | b0 | 1.465 | 0.062 | 23.53 | <0.0001 |
| | R2=0.77 | X1 | -0.007 | 0.003 | -2.44 | 0.0152 |
| | | X2 | 0.011 | 0.001 | 15.66 | <0.0001 |
| | | X12 | -0.005 | 0.001 | -4.29 | <0.0001 |
| | | X1X2 | 0.000 | 0.000 | -0.22 | 0.8295 |
| | | X22 | -0.001 | 0.000 | -15.20 | <0.0001 |
| | | X3(Blue) | 0.131 | 0.019 | 6.73 | <0.0001 |
| | | X3(Green) | 0.213 | 0.017 | 12.33 | <0.0001 |
| | | X3(Red) | -0.365 | 0.021 | -17.11 | <0.0001 |
