# Supplementary material for: Sexual Difference in the Optimum Environmental Conditions for Growth and Maturation of the Brown Alga Undaria pinnatifida in the Gametophyte Stage
Source: Genes (Basel). 2020 Aug 16;11(8):944. doi: 10.3390/genes11080944 (PMC7463851; doi:10.3390/genes11080944)
Supplement: Supplementary file 1 [file genes-11-00944-s001.zip › Supplemental Figures and Tables_REV.pptx]

## Slide 1
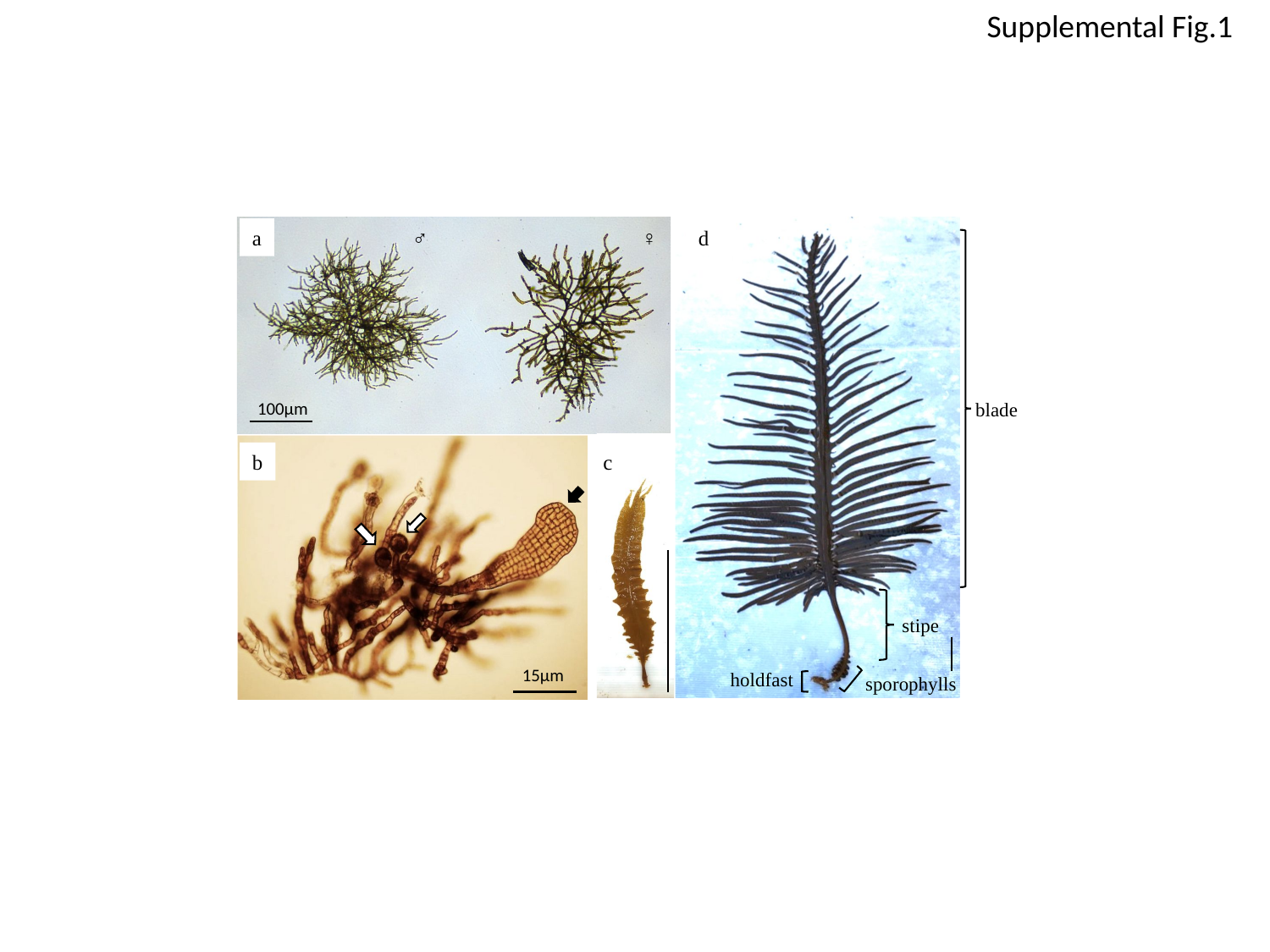

Supplemental Fig.1
♂
♀
d
a
blade
100μm
b
c
stipe
15μm
holdfast
sporophylls

## Slide 2
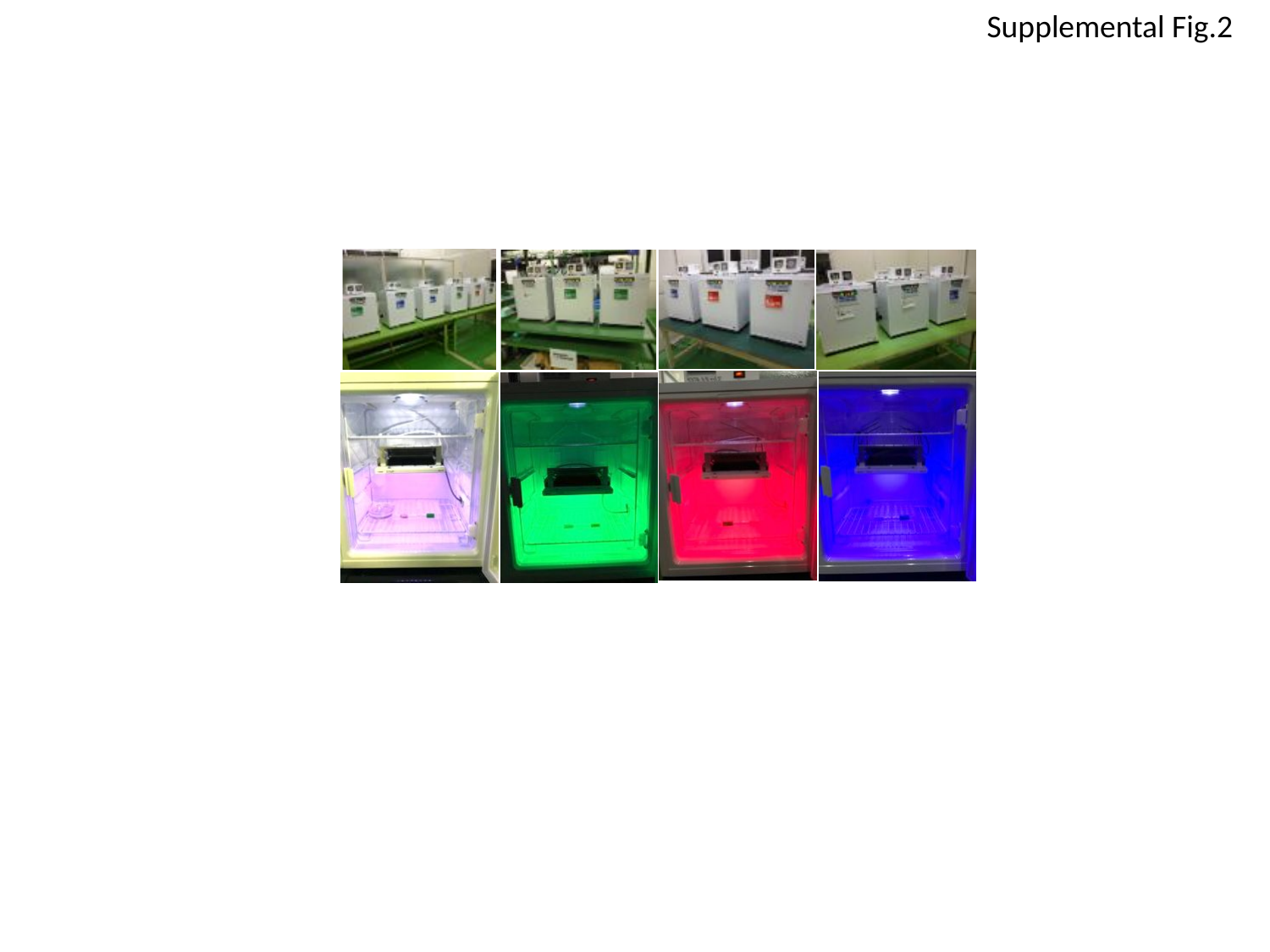

Supplemental Fig.2

## Slide 3
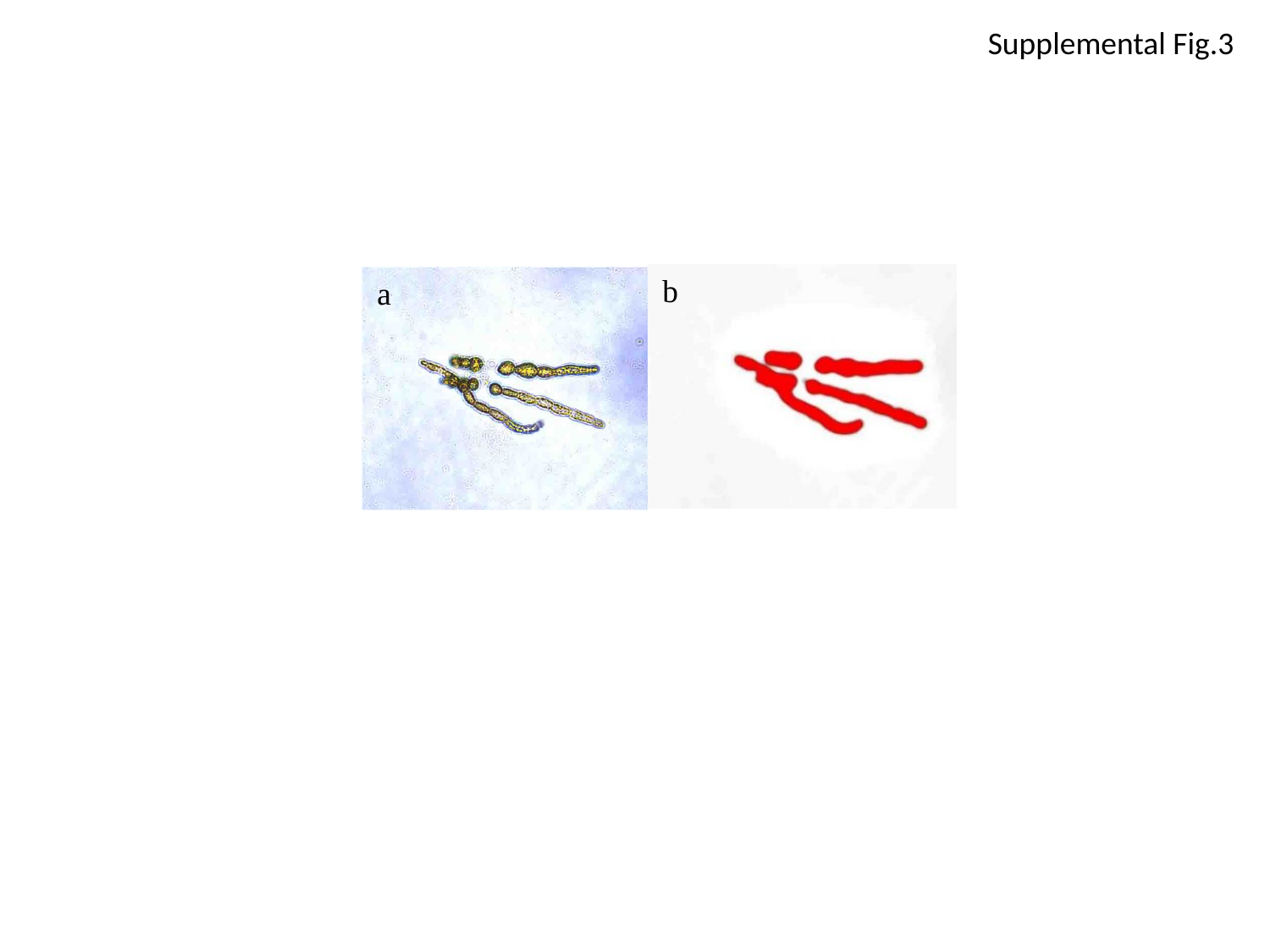

Supplemental Fig.3
b
a

## Slide 4
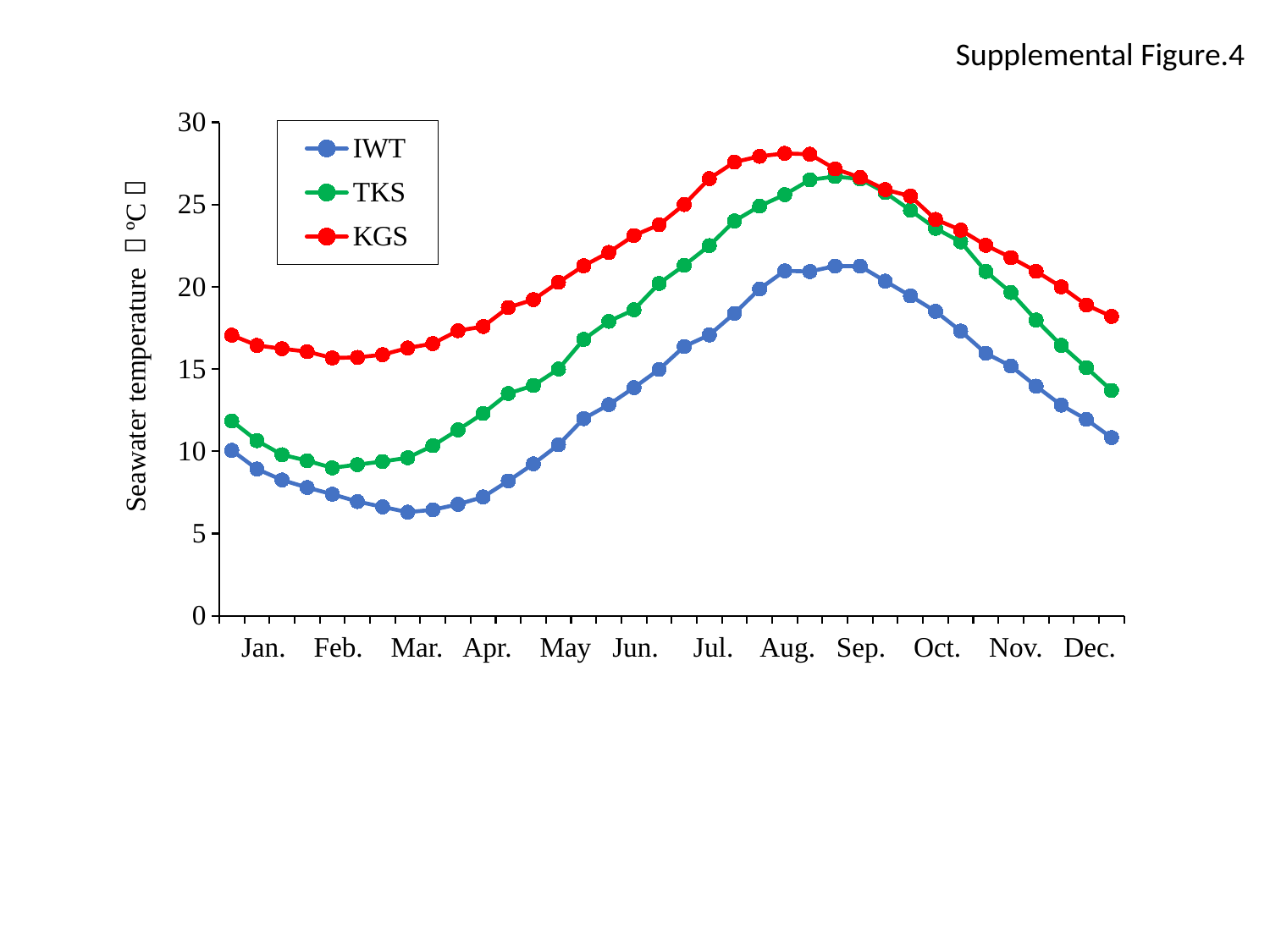

Supplemental Figure.4
### Chart
| Category | | | |
|---|---|---|---|
| 1 | 10.055444444444445 | 11.8408 | 17.057499999999997 |
| 2 | 8.919185185185185 | 10.641500000000002 | 16.4375 |
| 3 | 8.252 | 9.790545454545455 | 16.235 |
| 4 | 7.793555555555557 | 9.4249 | 16.0575 |
| 5 | 7.386777777777777 | 8.9892 | 15.672499999999998 |
| 6 | 6.942749999999999 | 9.186444444444444 | 15.707986111111111 |
| 7 | 6.616699999999999 | 9.3753 | 15.870000000000001 |
| 8 | 6.2957 | 9.604899999999997 | 16.2775 |
| 9 | 6.441588888888889 | 10.342272727272727 | 16.5475 |
| 10 | 6.773799999999999 | 11.296899999999999 | 17.3325 |
| 11 | 7.220055555555557 | 12.299000000000001 | 17.585 |
| 12 | 8.193777777777777 | 13.510500000000002 | 18.740000000000002 |
| 13 | 9.23079012345679 | 14.0 | 19.2175 |
| 14 | 10.393518518518517 | 15.0 | 20.2725 |
| 15 | 11.980281249999999 | 16.8 | 21.275 |
| 16 | 12.829660714285716 | 17.9 | 22.087500000000002 |
| 17 | 13.869291666666667 | 18.6 | 23.115000000000002 |
| 18 | 14.975000000000001 | 20.2 | 23.767500000000002 |
| 19 | 16.367375 | 21.3 | 25.0 |
| 20 | 17.071125 | 22.5 | 26.572499999999998 |
| 21 | 18.381625 | 24.0 | 27.58 |
| 22 | 19.872125 | 24.9 | 27.93 |
| 23 | 20.967055555555557 | 25.6 | 28.11 |
| 24 | 20.927 | 26.5 | 28.055 |
| 25 | 21.260406250000003 | 26.7 | 27.165000000000003 |
| 26 | 21.251125000000002 | 26.55 | 26.6475 |
| 27 | 20.340249999999997 | 25.72 | 25.905 |
| 28 | 19.446125000000002 | 24.650000000000002 | 25.509999999999998 |
| 29 | 18.50725 | 23.55 | 24.095 |
| 30 | 17.305861111111113 | 22.73 | 23.445 |
| 31 | 15.956583333333336 | 20.930000000000003 | 22.5225 |
| 32 | 15.178652777777778 | 19.65 | 21.775 |
| 33 | 13.958083333333335 | 17.98 | 20.94 |
| 34 | 12.8036875 | 16.440000000000005 | 19.9925 |
| 35 | 11.934263888888887 | 15.09 | 18.892500000000002 |
| 36 | 10.829250000000002 | 13.7 | 18.1975 |Seawater temperature （ºC）
Jan. Feb. Mar. Apr. May Jun. Jul. Aug. Sep. Oct. Nov. Dec.

## Slide 5
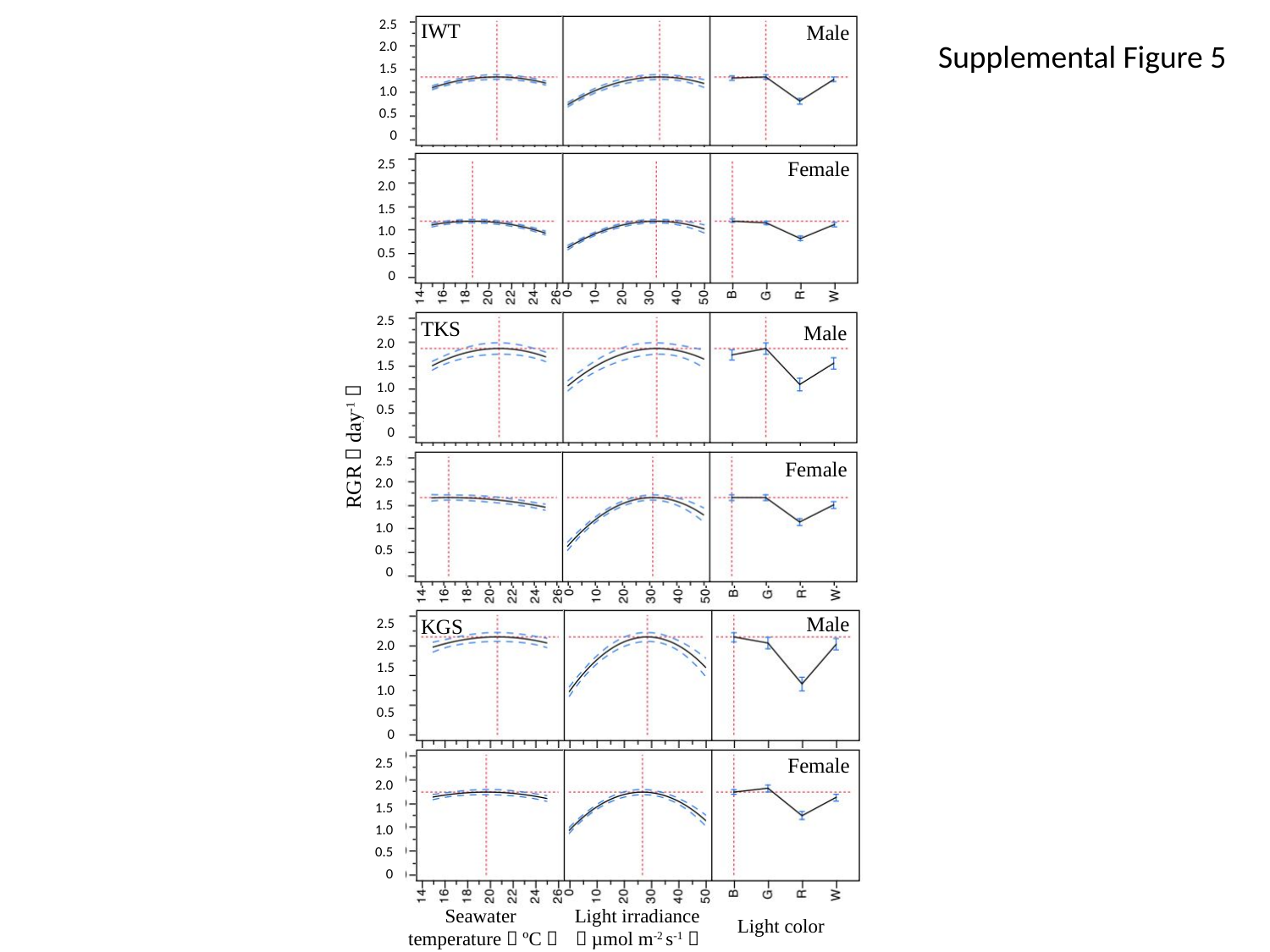

2.5
2.0
1.5
1.0
0.5
0
IWT
Male
Supplemental Figure 5
2.5
2.0
1.5
1.0
0.5
0
Female
2.5
2.0
1.5
1.0
0.5
0
TKS
Male
RGR（day-1）
2.5
2.0
1.5
1.0
0.5
0
Female
2.5
2.0
1.5
1.0
0.5
0
Male
KGS
2.5
2.0
1.5
1.0
0.5
0
Female
Seawater
temperature（ºC）
Light irradiance
（µmol m-2 s-1）
Light color

## Slide 6
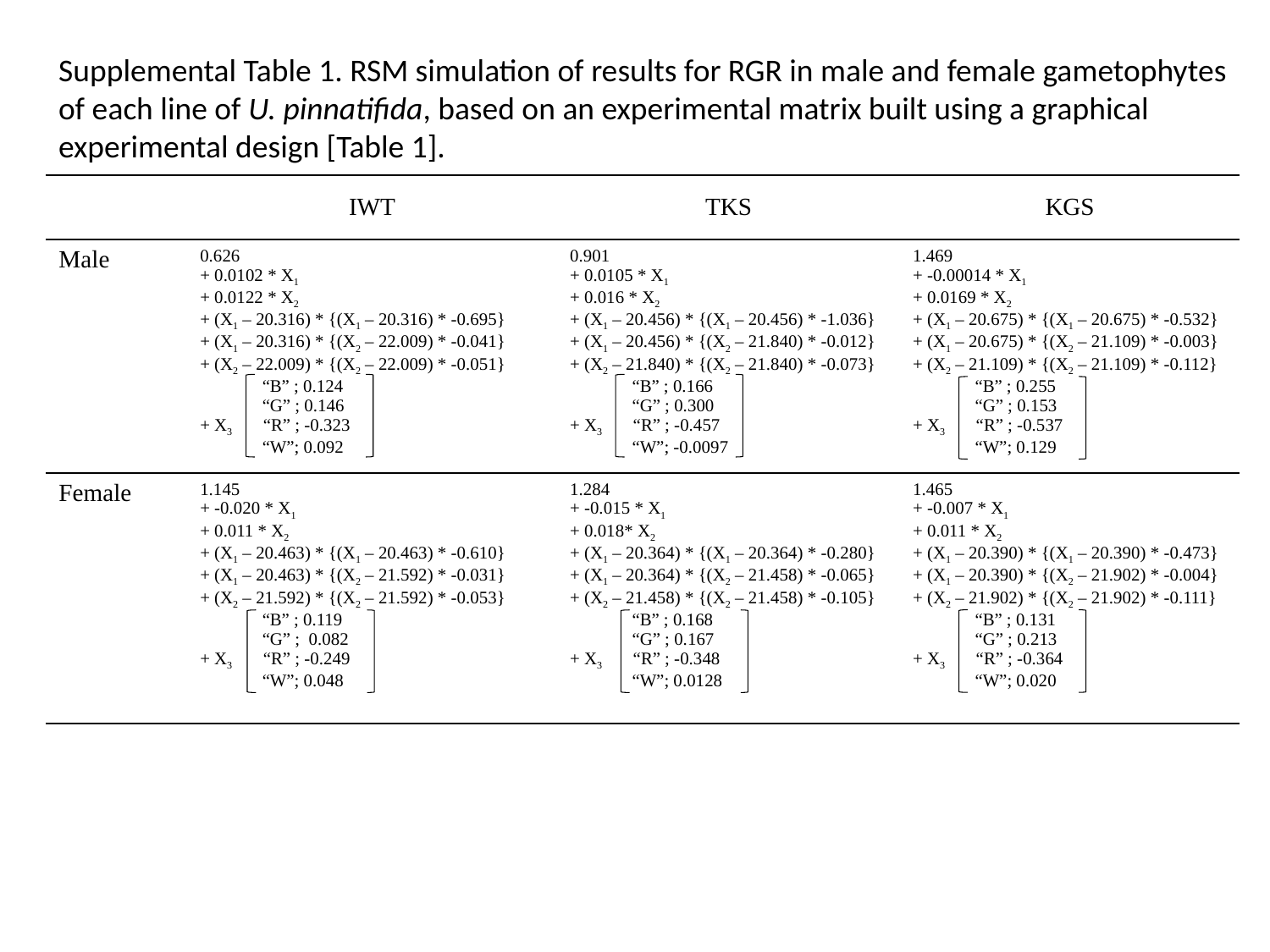

Supplemental Table 1. RSM simulation of results for RGR in male and female gametophytes of each line of U. pinnatifida, based on an experimental matrix built using a graphical experimental design [Table 1].
| | IWT | TKS | KGS |
| --- | --- | --- | --- |
| Male | 0.626 + 0.0102 \* X1 + 0.0122 \* X2 + (X1 – 20.316) \* {(X1 – 20.316) \* -0.695} + (X1 – 20.316) \* {(X2 – 22.009) \* -0.041} + (X2 – 22.009) \* {(X2 – 22.009) \* -0.051} “B” ; 0.124 “G” ; 0.146 + X3 “R” ; -0.323 “W”; 0.092 | 0.901 + 0.0105 \* X1 + 0.016 \* X2 + (X1 – 20.456) \* {(X1 – 20.456) \* -1.036} + (X1 – 20.456) \* {(X2 – 21.840) \* -0.012} + (X2 – 21.840) \* {(X2 – 21.840) \* -0.073} “B” ; 0.166 “G” ; 0.300 + X3 “R” ; -0.457 “W”; -0.0097 | 1.469 + -0.00014 \* X1 + 0.0169 \* X2 + (X1 – 20.675) \* {(X1 – 20.675) \* -0.532} + (X1 – 20.675) \* {(X2 – 21.109) \* -0.003} + (X2 – 21.109) \* {(X2 – 21.109) \* -0.112} “B” ; 0.255 “G” ; 0.153 + X3 “R” ; -0.537 “W”; 0.129 |
| Female | 1.145 + -0.020 \* X1 + 0.011 \* X2 + (X1 – 20.463) \* {(X1 – 20.463) \* -0.610} + (X1 – 20.463) \* {(X2 – 21.592) \* -0.031} + (X2 – 21.592) \* {(X2 – 21.592) \* -0.053} “B” ; 0.119 “G” ; 0.082 + X3 “R” ; -0.249 “W”; 0.048 | 1.284 + -0.015 \* X1 + 0.018\* X2 + (X1 – 20.364) \* {(X1 – 20.364) \* -0.280} + (X1 – 20.364) \* {(X2 – 21.458) \* -0.065} + (X2 – 21.458) \* {(X2 – 21.458) \* -0.105} “B” ; 0.168 “G” ; 0.167 + X3 “R” ; -0.348 “W”; 0.0128 | 1.465 + -0.007 \* X1 + 0.011 \* X2 + (X1 – 20.390) \* {(X1 – 20.390) \* -0.473} + (X1 – 20.390) \* {(X2 – 21.902) \* -0.004} + (X2 – 21.902) \* {(X2 – 21.902) \* -0.111} “B” ; 0.131 “G” ; 0.213 + X3 “R” ; -0.364 “W”; 0.020 |

## Slide 7
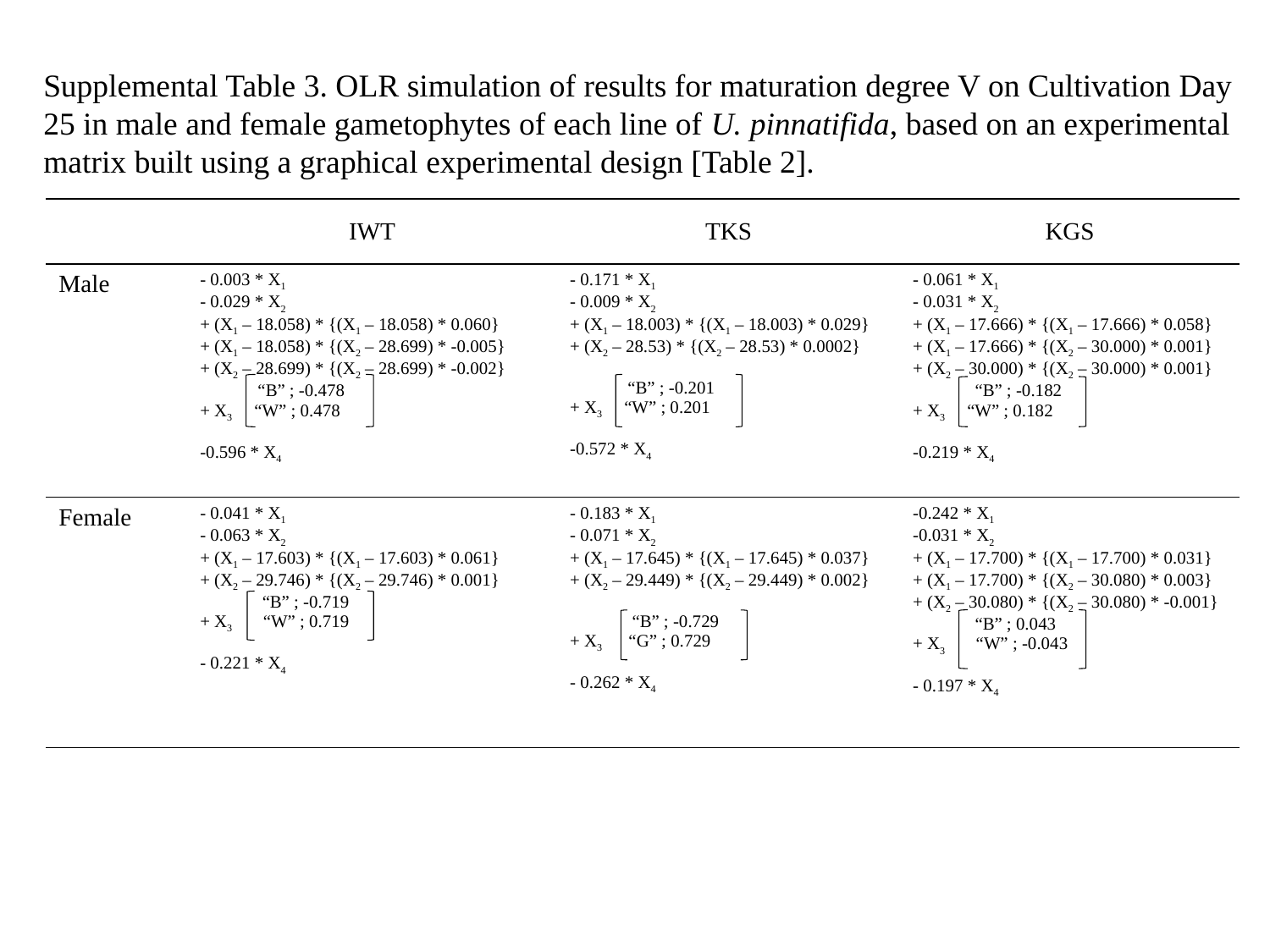

Supplemental Table 3. OLR simulation of results for maturation degree V on Cultivation Day 25 in male and female gametophytes of each line of U. pinnatifida, based on an experimental matrix built using a graphical experimental design [Table 2].
| | IWT | TKS | KGS |
| --- | --- | --- | --- |
| Male | - 0.003 \* X1 - 0.029 \* X2 + (X1 – 18.058) \* {(X1 – 18.058) \* 0.060} + (X1 – 18.058) \* {(X2 – 28.699) \* -0.005} + (X2 – 28.699) \* {(X2 – 28.699) \* -0.002} “B” ; -0.478 + X3 “W” ; 0.478 -0.596 \* X4 | - 0.171 \* X1 - 0.009 \* X2 + (X1 – 18.003) \* {(X1 – 18.003) \* 0.029} + (X2 – 28.53) \* {(X2 – 28.53) \* 0.0002} “B” ; -0.201 + X3 “W” ; 0.201 -0.572 \* X4 | - 0.061 \* X1 - 0.031 \* X2 + (X1 – 17.666) \* {(X1 – 17.666) \* 0.058} + (X1 – 17.666) \* {(X2 – 30.000) \* 0.001} + (X2 – 30.000) \* {(X2 – 30.000) \* 0.001} “B” ; -0.182 + X3 “W” ; 0.182 -0.219 \* X4 |
| Female | - 0.041 \* X1 - 0.063 \* X2 + (X1 – 17.603) \* {(X1 – 17.603) \* 0.061} + (X2 – 29.746) \* {(X2 – 29.746) \* 0.001} “B” ; -0.719 + X3 “W” ; 0.719 - 0.221 \* X4 | - 0.183 \* X1 - 0.071 \* X2 + (X1 – 17.645) \* {(X1 – 17.645) \* 0.037} + (X2 – 29.449) \* {(X2 – 29.449) \* 0.002} “B” ; -0.729 + X3 “G” ; 0.729 - 0.262 \* X4 | -0.242 \* X1 -0.031 \* X2 + (X1 – 17.700) \* {(X1 – 17.700) \* 0.031} + (X1 – 17.700) \* {(X2 – 30.080) \* 0.003} + (X2 – 30.080) \* {(X2 – 30.080) \* -0.001} “B” ; 0.043 + X3 “W” ; -0.043 - 0.197 \* X4 |

## Slide 8
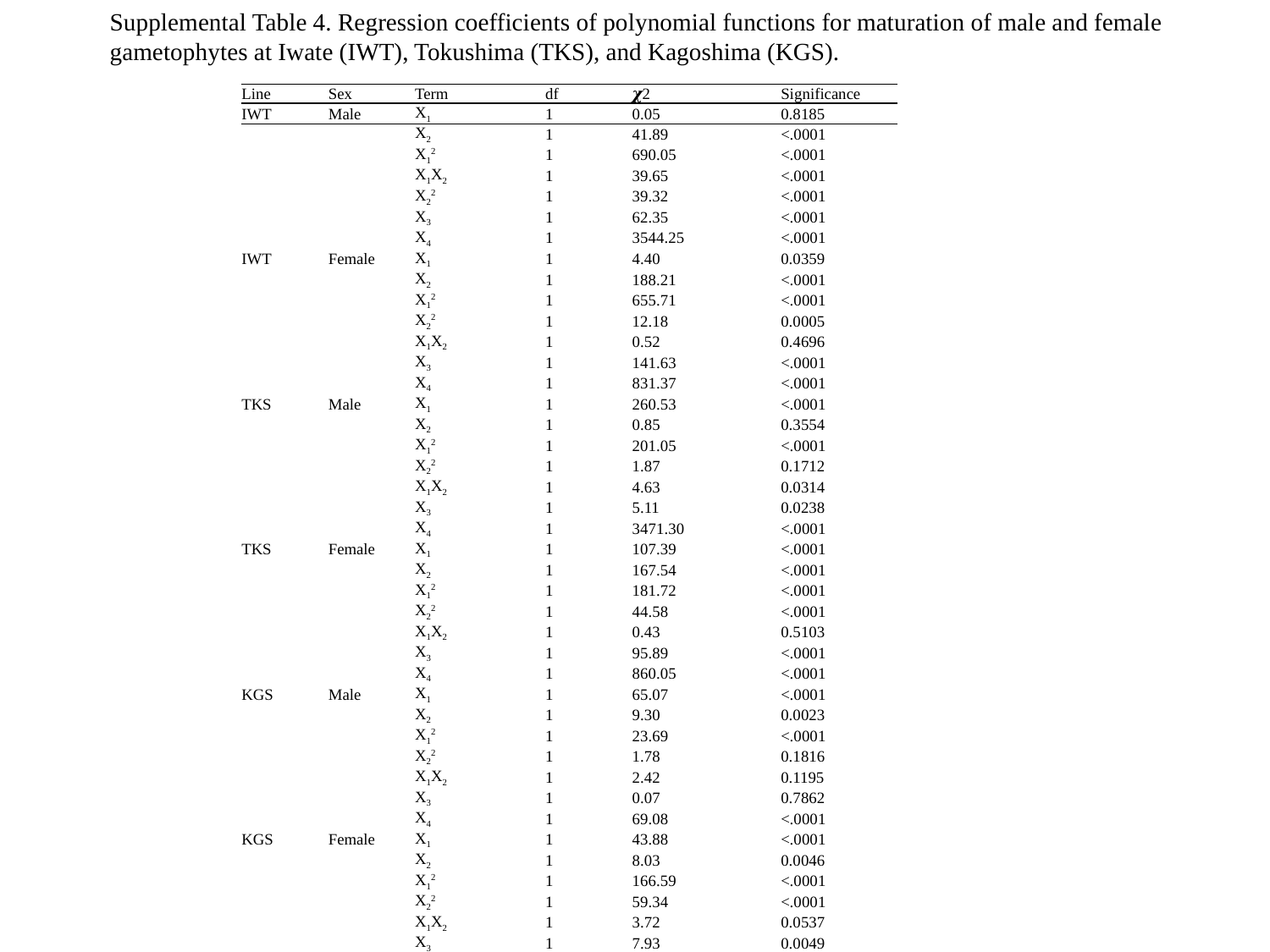

Supplemental Table 4. Regression coefficients of polynomial functions for maturation of male and female gametophytes at Iwate (IWT), Tokushima (TKS), and Kagoshima (KGS).
| Line | Sex | Term | df | 𝝌2 | Significance |
| --- | --- | --- | --- | --- | --- |
| IWT | Male | X1 | 1 | 0.05 | 0.8185 |
| | | X2 | 1 | 41.89 | <.0001 |
| | | X12 | 1 | 690.05 | <.0001 |
| | | X1X2 | 1 | 39.65 | <.0001 |
| | | X22 | 1 | 39.32 | <.0001 |
| | | X3 | 1 | 62.35 | <.0001 |
| | | X4 | 1 | 3544.25 | <.0001 |
| IWT | Female | X1 | 1 | 4.40 | 0.0359 |
| | | X2 | 1 | 188.21 | <.0001 |
| | | X12 | 1 | 655.71 | <.0001 |
| | | X22 | 1 | 12.18 | 0.0005 |
| | | X1X2 | 1 | 0.52 | 0.4696 |
| | | X3 | 1 | 141.63 | <.0001 |
| | | X4 | 1 | 831.37 | <.0001 |
| TKS | Male | X1 | 1 | 260.53 | <.0001 |
| | | X2 | 1 | 0.85 | 0.3554 |
| | | X12 | 1 | 201.05 | <.0001 |
| | | X22 | 1 | 1.87 | 0.1712 |
| | | X1X2 | 1 | 4.63 | 0.0314 |
| | | X3 | 1 | 5.11 | 0.0238 |
| | | X4 | 1 | 3471.30 | <.0001 |
| TKS | Female | X1 | 1 | 107.39 | <.0001 |
| | | X2 | 1 | 167.54 | <.0001 |
| | | X12 | 1 | 181.72 | <.0001 |
| | | X22 | 1 | 44.58 | <.0001 |
| | | X1X2 | 1 | 0.43 | 0.5103 |
| | | X3 | 1 | 95.89 | <.0001 |
| | | X4 | 1 | 860.05 | <.0001 |
| KGS | Male | X1 | 1 | 65.07 | <.0001 |
| | | X2 | 1 | 9.30 | 0.0023 |
| | | X12 | 1 | 23.69 | <.0001 |
| | | X22 | 1 | 1.78 | 0.1816 |
| | | X1X2 | 1 | 2.42 | 0.1195 |
| | | X3 | 1 | 0.07 | 0.7862 |
| | | X4 | 1 | 69.08 | <.0001 |
| KGS | Female | X1 | 1 | 43.88 | <.0001 |
| | | X2 | 1 | 8.03 | 0.0046 |
| | | X12 | 1 | 166.59 | <.0001 |
| | | X22 | 1 | 59.34 | <.0001 |
| | | X1X2 | 1 | 3.72 | 0.0537 |
| | | X3 | 1 | 7.93 | 0.0049 |
| | | X4 | 1 | 296.00 | <.0001 |
